# Building a completely positive factorization

**DOI:** 10.1007/s10100-017-0499-2

**Published:** 2017-11-15

**Authors:** Immanuel M. Bomze

**Affiliations:** ISOR and VCOR, University of Vienna, Vienna, Austria

**Keywords:** Copositive optimization, cp-rank, Schur complement

## Abstract

A symmetric matrix of order *n* is called *completely positive * if it has a symmetric factorization by means of a rectangular matrix with *n* columns and no negative entries (a so-called *cp factorization*), i.e., if it can be interpreted as a Gram matrix of *n* directions in the positive orthant of another Euclidean space of possibly different dimension. Finding this factor therefore amounts to angle packing and finding an appropriate embedding dimension. Neither the embedding dimension nor the directions may be unique, and so many cp factorizations of the same given matrix may coexist. Using a bordering approach, and building upon an already known cp factorization of a principal block, we establish sufficient conditions under which we can extend this cp factorization to the full matrix. Simulations show that the approach is promising also in higher dimensions.

## Introduction

A symmetric matrix is called *completely positive,* if it admits a symmetric rectangular matrix factorization with no negative entries; Berman and Shaked-Monderer (2003) is a monograph which focuses on linear-algebraic and graph theoretic properties of this matrix class. The concept—the notion was probably coined by Hall (1963), see also Diananda (1962)—had its origins from applications in combinatorics (block designs, Hall 1963). Further fields of application include physics, biology and statistics (Markovian models of DNA evolution, Kelly 1994), project management (stochastic and robust optimization, Natarajan 2011), and economic modeling (see Gray and Wilson 1980), and in recent years optimization applications became increasingly important.

We need some notation. By ${\mathbb {R}}^n$ we denote *n*-dimensional Euclidean space, by ${\mathbb {R}}^n_+$ its positive orthant, (column) vectors $\mathbf {v}\in {\mathbb {R}}^n$ are always in boldface while $\mathbf {v}^\top$ denotes their transpose (rows). The zero matrix of appropriate size is always denoted by *O*, and $A\le O$ for a matrix *A* of the same size means that no entry $A_{ij}$ is positive, while $A\le B$ means $A-B\le O$. $I_d$ denotes the $d\times d$ identity matrix, with its *i*th column $\mathbf {e}_i\in {\mathbb {R}}^d$. For a scalar *t*, we denote by $t_+ = \max \left\{ 0,t \right\}$ while for a matrix *A* we put $A_+ = [(A_{ij})_+]_{i,j}$. Further, we designate by $A^{\bullet 2} = [(A_{ij})^2]_{i,j}$ the Hadamard square of *A*. The cone of all symmetric positive-semidefinite matrices of some fixed order is denoted by ${\mathcal {P}}$, and the cone of all symmetric matrices with no negative entries by ${\mathcal {N}}$. Finally, let ${X}^{1/2}\in {\mathcal {P}}$ denote the symmetric square-root of a matrix $X\in {\mathcal {P}}$. Note that even if $X\ge O$, we may have negative entries in $X^{1/2}$. If however ${X}^{1/2}\ge O$, then *X* belongs to the the cone ${\mathcal {C}}$ of all (symmetric) completely positive matrices

$$\begin{aligned} {\mathcal {C}}= \left\{ X\in {\mathcal {P}}: X = F^\top F \text{ for } \text{ some } \text{ possibly } \text{ rectangular } \text{ matrix } F\ge O \right\} \,. \end{aligned}$$

We call $X=F^\top F$ a *completely positive (cp) factorization.*

One immediate geometric interpretation is as follows: write $F=[{\mathbf {f}}_1\, \ldots , {\mathbf {f}}_n]$ with ${\mathbf {f}}_i\in {\mathbb {R}}^m_+$. Then $X_{ij} = {\mathbf {f}}_i^\top {\mathbf {f}}_j$ for all *i*, *j*, so *X* is the Gram matrix of the directions ${\mathbf {f}}_i$. In other words, finding *F* (and *m*) amounts to find a space ${\mathbb {R}}^m$ and directions in its positive orthant ${\mathbb {R}}^m_+$ such that *X* describes (length and) angles of this direction, i.e. solving an *angle packing problem*.

The minimal number of rows in *F* yielding a cp factorization of *X* is called the *cp-rank* of *X*. In light of above interpretation, determining the cp-rank means to find the smallest embedding dimension such that the angle packing problem has a solution. This embedding dimension, i.e., the cp-rank can exceed the order *n* of *X* (i.e., the number of directions). But the cp-rank is bounded by ${n+1\atopwithdelims ()2}-4 \sim \frac{n^2}{2}$, and this bound is asymptotically tight (Bomze et al. 2015; Shaked-Monderer et al. 2015), refuting a conjecture suggesting $n^2/4$ published 20 years ago (Drew et al. 1994).

An alternative format of cp factorization can be obtained using $F^\top$ rather than *F*, i.e., writing

$$\begin{aligned} X=[\mathbf {x}_1, \ldots , \mathbf {x}_m]\, [\mathbf {x}_1, \ldots , \mathbf {x}_m]^\top = \sum _{i=1}^m \mathbf {x}_i\mathbf {x}_i^\top \end{aligned}$$

with $\mathbf {x}_i\in {\mathbb {R}}^n_+$. Thus, searching for a minimal cp factorization would amount searching for the shortest sum in above additive decomposition into rank-one matrices $\mathbf {x}_i\mathbf {x}_i^\top$ built upon non-negative vectors $\mathbf {x}_i\in {\mathbb {R}}^n_+$. With this algebraic approach, we may see why having such a cp factorization is important: suppose $X^*$ emerges as the solution of a copositive optimization problem (see below) which is a conic approximation or reformulation of, say, a non-convex quadratic optimization problem $z^*=\min \nolimits _{\mathbf {x}\in {\mathbb {R}}^n_+} \left\{ \mathbf {x}^\top Q \mathbf {x}: A\mathbf {x}= \mathbf {b} \right\}$ over a polyhedron. This is an NP-hard problem class. It turns out that under weak assumptions (Burer 2009), any of the vectors $\mathbf {x}_i$ from a rank-one summand $\mathbf {x}_i\mathbf {x}_i^\top$ occurring in a cp factorization of $X^*$ will be an optimal (or an approximate) solution to $z^*$.

So both representations have their advantages and can easily be transformed into each other. In the sequel, we will adhere to the format $X=F^\top F$ suggested by the angle packing interpretation. As indicated above, finding a cp factorization of a given matrix can yield good or even optimal solutions to hard optimization problems. Moreover, characteristics like embedding dimension for the angle packing problem will give important information on the geometry of the related conic problem. Recall that in any linear optimization problem over a convex set, the solution (if it exists) is attained at the boundary of the feasible set, and indeed all the complexity of the reformulated hard problems is shifted to the analysis of that boundary. However, unlike the boundary of the feasible sets for LPs and SDPs (both problem classes solvable in polynomial time to arbitrary accuracy), this boundary can contain matrices *X* of full rank and those with all entries strictly positive. The cp-rank and more general, any cp factorization of *X*, can give more information on *X* with respect to this geometrical structure and at the same time provide alternative (approximate) solutions. More detail will be provided in Sect. 2 below.

In this paper we aim at obtaining a cp factorization of a symmetric $(n+1)\times (n+1)$ matrix $Y=H^\top H$ by a bordering approach: we assume that we know a cp factorization for a principal $n\times n$ submatrix $X=F^\top F$ of *Y*, and derive sufficient conditions under which we can specify a suitable factor *H*. This is the content of Sects. 3 and 7. These sufficient conditions generalize and complement previous investigations of the same kind in Salce and Zanardo (1993), leading to a structurally different cp factorization (essentially, the role of a lower block-triangular factor in Salce and Zanardo (1993) is now played by an upper block-triangular one). In Sect. 4, we take an optimization-inspired approach, leading to LP- or QP-based relaxations of the main property, in order to systematically find out whether or not this new sufficient condition is satisfied. This approach also may enable us to efficiently search for constellations (i.e., selecting the bordering row) where the condition is met. A small empirical study provided in Sect. 5 shows that our approach is more promising. In Sect. 6 we show that our approach indeed suffices to obtain a cp factorization for all completely positive $3\times 3$ matrices, establishing in an elementary way the well-known fact that the cp-rank of these does not exceed three. This has been known before, but our approach seems less involved than the previous arguments. Inspired by this, we move on in Sect. 7 to discuss extensions in higher dimensions.

## Motivation and preprocessing

Since the explicit introduction of *copositive optimization* (or copositive programming) by Bomze et al. (2000), Quist et al. (1998), we observe a rapid evolution of this field. One reason for the success is culminating in the important paper (Burer 2009) where it is shown that every mixed-binary (fractional) quadratic optimization problem can be written as a copositive optimization problem, which is a linear optimization problem over the cone ${\mathcal {C}}$ subject to linear constraints, see Amaral and Bomze (2015), Amaral et al. (2014), Bomze and Jarre (2010) and Burer (2009), and recently many similar copositive representation results followed. For some surveys, we refer to Bomze (2012) Bomze et al. (2012), Burer (2012) and Dür (2010).

The terminology copositive optimization has its justification as the dual cone of ${\mathcal {C}}$ coincides with the cone of copositive matrices of the same order. Recall that a symmetric $n\times n$ matrix is said to be *copositive* if it generates a quadratic form taking no negative values over the positive orthant ${\mathbb {R}}^n_+$. The usual conic approximation algorithms for solving a copositive (or completely positive) optimization problem use (subsets of) the outer approximation ${\mathcal {P}}\cap {\mathcal {N}}$ of ${\mathcal {C}}$. However, often copositive optimization problems are used to reformulate hard (mixed-binary) quadratic optimization problems which in turn may encode combinatorial optimization problems (see Bomze et al. 2000; Burer 2009; Natarajan 2011; Quist et al. 1998 and references therein). The optimal solution of the latter is encoded by an $r\times n$ matrix *F* with no negative entries, in a cp factorization $X=F^\top F\in {\mathcal {C}}$.

Once we arrive at a solution $X\in {\mathcal {P}}\cap {\mathcal {N}}$ of the relaxation, we should try to find this *F*, not only to show that in this instance, the relaxation is exact, but also to retrieve the solution of the original problem (the so-called rounding procedure). Very few recent papers deal with approximating ${\mathcal {C}}$ from within: a theoretical characterization of interior points of ${\mathcal {C}}$ is presented in Dickinson (2010) and Dür and Still (2008), while algorithmic aspects of factorization are the focus of Jarre and Schmallowsky (2009).

Suppose that we want to find an explicit factorization of an $(n+1)\times (n+1)$ matrix *Y* which we scale such that

1 $$\begin{aligned} Y=\left[ {\begin{array}{ll} 1 &{}\quad \mathbf {x}^\top \\ \mathbf {x}&{}\quad X\\ \end{array}}\right] \, , \end{aligned}$$

building upon an already known cp factorization of the principal block *X*. Note that as ${\mathcal {C}}\subseteq {\mathcal {P}}$, we can immediately spare our efforts if one diagonal entry of *Y* is negative. Similarly, positive-semidefiniteness of *Y* implies that a zero diagonal entry forces the whole row and column to be zero, in which case we can remove it and continue with a smaller principal submatrix with strictly positive diagonal elements. In the end, we just have to enlarge the factor *H* by suitably adding zero columns, to obtain the original *Y*. Hence we may and do assume $Y_{ii}>0$ for all *i*. Next, we observe that with any positive-definite diagonal matrix $\Delta$ and any factorization $Y=H^\top H$, we get another one of $\Delta Y\Delta = (H\Delta )^\top (H\Delta )$ of the same size. As also $\Delta ^{-1}$ is positive-definite, we may use $\Delta _{ii} = Y_{ii}^{-1/2}>0$ and concentrate on the case where the diagonal of *Y* contains only unity entries. This is the starting point of (1).

Next we proceed as in Dickinson and Dür (2012) which offers an algorithmic procedure to obtain a minimal cp factorization, applied to *Y* with a special sparsity pattern. As a preliminary step, we check the necessary condition $Y\in {\mathcal {P}}\cap {\mathcal {N}}$; since $X\in {\mathcal {C}}\subseteq {\mathcal {P}}\cap {\mathcal {N}}$, we merely must check $\mathbf {x}\in {\mathbb {R}}^n_+$ to ensure $Y\in {\mathcal {N}}$; and $X-\mathbf {x}\mathbf {x}^\top \in {\mathcal {P}}$ to ensure $Y\in {\mathcal {P}}$, by Schur complementation. Note that positive-semidefiniteness of $Y\ge O$ and $Y_{ii}=1$ implies $Y_{ij}\in [0,1]$ for all *i*, *j*, therefore any possible cp factorization matrix *H* (i.e. satisfying $H^\top H=Y$) also must have entries between zero and one.

Suppose that $X\mathbf {v}= \mathbf {o}$ but $\mathbf {v}^\top \mathbf {x}\ne 0$; then we would arrive at the contradiction $0\le \mathbf {v}^\top (X-\mathbf {x}\mathbf {x}^\top )\mathbf {v}= - (\mathbf {v}^\top \mathbf {x})^2< 0$. So we may and do assume in the sequel that $\mathbf {x}\in (\text{ ker } X)^\perp$ or equivalently, that

2 $$\begin{aligned} XX^+ \mathbf {x}= \mathbf {x}\, , \end{aligned}$$

where $X^+$ denotes the *Moore/Penrose generalized inverse (MPGI)* of any matrix.

## Various cp factorization strategies

The cp factorization problem has received considerable attention as a special (symmetric) variant of the nowadays heavily researched *nonnegative matrix factorization (NMF)* problem. For a recent survey on NMF see, e.g. Wang and Zhang (2013). In this context, the cp factorization problem is also addressed as *Symmetric NMF*, e.g. in He et al. (2011) where a simple parallelizable iterative procedure is proposed which is shown to converge to a stationary point (not the global solution) of the (nonconvex) least squares approximation problem $\min \nolimits _H \Vert Y- H^\top H \Vert$, with application to probabilistic clustering in large instances. This article fits into the tradition of convergence analysis in Matheuristics, as performed masterly in Gutjahr (1995); see also Gutjahr (2010). In contrast to these approaches, we focus on finite, not on iterative methods, although possibly employing iterative solutions to (easy) subproblems. For many other approaches, we refer to Anstreicher and Burer (2010), Berman and Hershkowitz (1987), Berman and Xu (2004), Berman and Rothblum (2006), Dickinson and Dür (2012), Shaked-Monderer (2009), Shaked-Monderer (2013) and Sponsel and Dür (2014) and the recent review (Berman et al. 2015), to cite just a few. Another recent paper (Zhou and Fan 2015) deals with algorithmic strategies for factorization based upon conic optimization, for random instances of relatively moderate order $n\ge 10$.

The original cp factorization problem can also be seen directly: to obtain $Y=H^\top H$ with an $s\times (n+1)$ matrix $H\ge O$, solve a system of $n+2\atopwithdelims ()2$ quadratic equations in $s(n+1)$ nonnegative variables. As detailed above, we can bound only $s\le {{n+2}\atopwithdelims ()2}-4$ and hence need in the worst case $\frac{1}{2} (n^3+4n^2-3n+6)$ nonnegative variables, which from an algorithmic perspective is practically prohibitive even for small instances. As already announced, we here assume that we know the cp factorization

3 $$\begin{aligned} X= F^\top F\qquad \text{ where } \quad F \quad \text{ is } \text{ an } r\times n\text{-matrix } \text{ with } \text{ no } \text{ negative } \text{ entries. } \end{aligned}$$

Since $X_{ij}\in [0,1]$, also $F_{ij}\in [0,1]$ for all entries. As mentioned earlier, $\text{ cp-rank }(X)$ is defined as the smallest *r* such that (3) holds. Hence given any *F* satisfying (3), we always have $\text{ cp-rank }(X)\le r$, and since the cp-rank may also be of order $n^2$, we can have $r>n$ if $n>4$. See Bomze et al. (2014) for recent bounds in the range $n\le 11$ and Bomze et al. (2015) for all larger *n*.

Recall that for the $n\times r$ matrix $F^\top$, the MPGI is given by

$$\begin{aligned} (F^\top )^+ = F(F^\top F)^+ = FX^+\, . \end{aligned}$$

This concept enables us to study the linear equation system $F^\top \mathbf {y}= \mathbf {x}$ in $\mathbf {y}$. It always has a solution since $F^\top (F^\top )^+\mathbf {x}= F^\top F X^+\mathbf {x}= XX^+\mathbf {x}= \mathbf {x}$ by (2), and the general solution is of the form

4 $$\begin{aligned} \mathbf {y}= (F^\top )^+ \mathbf {x}+ (I_m - (F^\top )^+F^\top )\mathbf {u}= \mathbf {p}+ (I_m - FX^+ F^\top ) \mathbf {u}\, , \quad \mathbf {u}\in {\mathbb {R}}^m\, , \end{aligned}$$

where $\mathbf {p}=FX^+ \mathbf {x}$. The solution of minimum norm in (4) is $\mathbf {y}=\mathbf {p}$ (with $\mathbf {u}=\mathbf {o}$). Now $\mathbf {p}^\top \mathbf {p}= \mathbf {x}^\top X^+ F^\top F X^+ \mathbf {x} = \mathbf {x}^\top X^+ \mathbf {x}\le 1$, where the latter inequality follows from $X-\mathbf {x}\mathbf {x}^\top \in {\mathcal {P}}$. Hence there is always a solution $\mathbf {y}$ to $F^\top \mathbf {y}= \mathbf {x}$ with $\mathbf {y}^\top \mathbf {y}\le 1$. This was proved, e.g., in Salce and Zanardo (1993, Lem.1.1,Cor.1.3). Sometimes $\mathbf {y}=\mathbf {p}$ is the only choice, but for $r>n$ there could be better choices, see below. If $\mathbf {p}$ happens to have no negative entries, *Y* is said to have the *property of positivity of least squares solution (PLSS)* in Berman and Shaked-Monderer (2003, pp.98ff). PLSS ensures that the lower triangular block factorization reviewed in Sect. 3.1 below works, but this property is quite restrictive as will be documented by our empirical study. Anyhow, if *X* is diagonal as in a related article on cp factorization (Kalofolias and Gallopoulos 2012), PLSS holds: obviously $F={X}^{1/2} \ge O$ provides a cp factorization with $\mathbf {p}=FX^+\mathbf {x}\in {\mathbb {R}}^n_+$.

### Lower triangular blocks

Complete positivity of *Y* as in (1) is characterized in (Salce and Zanardo 1993, Prop.1.4) as follows (with a slight change of notation): there is an $r\times n$ matrix $F_0$ with no negative entries, and a vector $\mathbf {y}_0\in {\mathbb {R}}^r_+$ with $\mathbf {y}_0^\top \mathbf {y}_0 =1$ such that $F_0^\top \mathbf {y}_0 = \mathbf {x}$ and $X= F_0^\top F_0$.

Since completely positive factorizations are by no means unique, knowledge of *F* in (3) does not imply that the above $F_0$ and $\mathbf {y}_0$ are known. In particular, it is not guaranteed that $F=F_0$ or $\mathbf {y}=\mathbf {y}_0$. They can have even different sizes.

If we would like to search for $(F_0,\mathbf {y}_0)$ directly, this amounts to solving a system of, again, ${n+1\atopwithdelims ()2}+ n+1= {n+2\atopwithdelims ()2}$ quadratic equations in now $(r+1)n\le \frac{1}{2} (n^3+n^2-6n)$ nonnegative variables, slightly less than the original problem but still prohibitively demanding.

Anyhow, assume now that there is a *nonnegative* solution $\mathbf {y}\in {\mathbb {R}}^r_+$ to $F^\top \mathbf {y}= \mathbf {x}$ with $\mathbf {y}^\top \mathbf {y}\le 1$. Then we can use factors *H* with lower block triangular structure as follows:

5 $$\begin{aligned} Y= H^\top H \qquad \text{ with }\qquad H= \left[ \begin{array}{ll} \beta &{}\quad \mathbf {o}^\top \\ \mathbf {y}&{}\quad F\\ \end{array}\right] \quad \text{ where }\quad \beta = \sqrt{1-\mathbf {y}^\top \mathbf {y}}\, . \end{aligned}$$

This can be checked by straightforward calculation. From an algorithmic perspective it could pay to first try (5), e.g. by solving the linear optimization problem

6 $$\begin{aligned} \min \left\{ \sum _i y_i : F^\top \mathbf {y}= \mathbf {x}\, ,\, \mathbf {y}\in {\mathbb {R}}^m_+ \right\} \, , \end{aligned}$$

or even the convex QP variant with an objective $\mathbf {y}^\top \mathbf {y}$. Since *H* has one more row than *F*, the cp-rank increment from *X* to *Y* cannot exceed one, if *F* provides a minimal cp factorization. Moreover, if in this situation $\mathbf {y}^\top \mathbf {y}=1$, then the cp-rank of *Y* is even equal to that of *X*, as observed in Berman and Shaked-Monderer (2003, Exerc. 3.7, p.146).

### Upper triangular blocks

Unfortunately, unless $F=F_0$ by chance, problem (6) can be infeasible or its feasible set can have empty intersection with the unit ball. So we will propose an alternative approach where we can allow for factorizations $X=F^\top F$ and vectors $\mathbf {y}\in {\mathbb {R}}^r$ such that $F^\top \mathbf {y}= \mathbf {x}$, where some entries of $\mathbf {y}$ may be negative. In this case, there are always solutions inside the unit ball, as detailed above after (4).

#### Theorem 3.1

For $0\le \alpha \le 1$ denote by $\varphi (\alpha ) = 1 + \sqrt{1-\alpha }$. Suppose $F^\top \mathbf {y}= \mathbf {x}$ with $\mathbf {y}^\top \mathbf {y}\le 1$ and such that

7 $$\begin{aligned} \mathbf {y}\mathbf {x}^\top \le \varphi (\mathbf {y}^\top \mathbf {y}) F\, , \end{aligned}$$

where $\le$ is understood entrywise. Then

- $\quad G:= {[I_r - \mathbf {y}\mathbf {y}^\top ]}^{1/2} F \quad$ is an $r\times n$ matrix with no negative entries, and
- 8 $$\begin{aligned} Y= H^\top H \qquad \text{ with }\qquad H= \left[ \begin{array}{ll} 1 &{}\quad \mathbf {x}^\top \\ \mathbf {o}&{}\quad G\\ \end{array}\right] \end{aligned}$$
  gives an explicit completely positive factorization of *Y*.

#### Proof

It is easy to verify that $I_r - \mathbf {y}\mathbf {y}^\top \in {\mathcal {P}}$ and that

9 $$\begin{aligned} {[I_m - \mathbf {y}\mathbf {y}^\top ]}^{1/2} = I_m - \frac{1}{\varphi (\mathbf {y}^\top \mathbf {y})} \, \mathbf {y}\mathbf {y}^\top \, . \end{aligned}$$

So $\varphi (\mathbf {y}^\top \mathbf {y})G = \varphi (\mathbf {y}^\top \mathbf {y}) F - \mathbf {y}(F^\top \mathbf {y})^\top = \varphi (\mathbf {y}^\top \mathbf {y})F- \mathbf {y}\mathbf {x}^\top$, and (a) follows. The matrix product in (b) equals

$$\begin{aligned} \left[ {\begin{array}{ll} 1 &{}\quad \mathbf {x}^\top \\ \mathbf {x}&{}\quad \mathbf {x}\mathbf {x}^\top + G^\top G\\ \end{array}}\right] \, , \end{aligned}$$

and the lower right block is $\mathbf {x}\mathbf {x}^\top + F^\top (I_m - \mathbf {y}\mathbf {y}^\top ) F = F^\top F = X$, so the product is indeed *Y*. $\square$

For $\mathbf {x}=\mathbf {o}$, condition (7) is trivially satisfied, and (8) and (5) coincide. But only (7) also still holds for small $\max _j x_j >0$ and fixed positive *F*, whereas (6) could be immediately rendered infeasible even for small positive departures of $\mathbf {x}$ from $\mathbf {o}$, because feasibility of (6) is a homogeneous property; notice that from the 1600 cases generated in Sect. 5, only 9 satisfied $\mathbf {y}\ge \mathbf {o}$. This would not hurt for other purposes, e.g. Natarajan and Teo (2017) aiming at a more general factorization, but for a lower triangular factorization (5) it is essential. So only Theorem 3.1 may be viewed as a quantitative perturbation result, dealing with departures from the trivial block-diagonal case $\mathbf {x}=\mathbf {o}$.

As mentioned, cp factorizations need not be unique, and with respect to some criteria, the one proposed above need not be optimal. However, if the cp factorization of *X* was already (close to) minimal, then also above factorization is (close to) minimal, because the increment of embedding dimension for the angle packing problem is one. Section 7 below presents strategies to increase this increment, but for staying close to minimal, above strategy should be tried first.

The next section deals with condition (7). We will specify algorithmic approaches to satisfying this condition, and also show how to obtain an explicit factorization for all completely $3\times 3$ matrices in this way.

## En route to satisfying (7)

The function $\varphi$ as defined in Theorem 3.1 is decreasing, concave, and satisfies $\varphi (0)=2$ as well as $\varphi (1)=1$. Therefore we have the following estimates

10 $$\begin{aligned} 1\le 2-\alpha \le \varphi (\alpha ) \quad \text{ for } \text{ all }\,\alpha \in [0,1]\, . \end{aligned}$$

The approximation of lowest order uses the constant underestimation in (10), which results in linear constraints: so, any $\mathbf {y}\in {\mathbb {R}}^m$ with

11 $$\begin{aligned} \left. {\begin{array}{lll} F^\top \mathbf {y}&{}= &{}\mathbf {x}\\ \mathbf {y}^\top \mathbf {y}&{}\le &{}1\\ \mathbf {y}\mathbf {x}^\top &{}\le &{}F\\ \end{array}} \right\} \end{aligned}$$

satisfies (7). The first-order approximation uses the linear underestimation in (10). This yields (inhomogeneous) convex quadratic constraints:

12 $$\begin{aligned} \left. {\begin{array}{lll}F^\top \mathbf {y}&{}= &{}\mathbf {x}\\ \mathbf {y}^\top \mathbf {y}&{}\le &{}1\\ \mathbf {y}\mathbf {x}^\top &{}\le &{}(2-\mathbf {y}^\top \mathbf {y}) F\\ \end{array}} \right\} \, . \end{aligned}$$

Likewise, (12) implies (7). Finally, we can rewrite (7) without square roots: it is evident by elementary calculations that

13 $$\begin{aligned} \left. {\begin{array}{rll} F^\top \mathbf {y}&{}= &{}\mathbf {x}\\ \mathbf {y}^\top \mathbf {y}&{}\le &{}1\\ \left( \left[ \mathbf {y}\mathbf {x}^\top - F\right] _+\right) ^{\bullet 2} &{}\le &{}(1-\mathbf {y}^\top \mathbf {y}) F^{\bullet 2}\\ \end{array}} \right\} \end{aligned}$$

is equivalent to (7). As mentioned above, only the last conditions in (11), (12) or (13) can be violated by $\mathbf {y}=\mathbf {p}$. To increase our chances of satisfying (7), we therefore employ an optimization approach in that we allow for $\mathbf {y}=\mathbf {p}+ P\mathbf {u}$ as in (4) with

14 $$\begin{aligned} P= I_r -FX^+F^\top \end{aligned}$$

the orthoprojector onto $\text{ ker } (F^\top )= (\mathrm{im }F)^\perp$. Now $\mathbf {y}^\top \mathbf {y}\le 1$ is no longer guaranteed, but we know by $\mathbf {p}= FX^+\mathbf {x}\perp P\mathbf {u}$ and $P^\top P = P$ that

15 $$\begin{aligned} \mathbf {y}^\top \mathbf {y}=\mathbf {p}^\top \mathbf {p}+ \mathbf {u}^\top P \mathbf {u} \, , \end{aligned}$$

and (Salce and Zanardo 1993, Lem. 1.1,Cor. 1.3) guarantees that at least $\mathbf {p}^\top \mathbf {p}\le 1$. Hence we consider the optimization problems

$$\begin{aligned} \min \left\{ \mathbf {y}^\top \mathbf {y}: \mathbf {y}\in {\mathbb {R}}^r \,, \, \mathbf {y} \text{ satisfies } ~(\triangle ) \right\} \, , \end{aligned}$$

where $(\triangle )$ stands for (11), (12) or (13).

### Proposition 4.1

Suppose that $\mathbf {x}\ne \mathbf {o}$ in a given factorization (3) for (1).

(a1) Consider the convex, linearly constrained QP

16 $$\begin{aligned} \pi _0 = \min \left\{ \mathbf {y}^\top \mathbf {y}: \mathbf {y}\in {\mathbb {R}}^r \, , \, F^\top \mathbf {y}=\mathbf {x}\, , \, \mathbf {y}\mathbf {x}^\top \le F \right\} \, . \end{aligned}$$

Then

1. any feasible $\mathbf {y}$ with $\mathbf {y}\ge \mathbf {o}$ satisfies $\mathbf {y}^\top \mathbf {y}\le 1$;
2. any feasible $\mathbf {y}$ with $\mathbf {y}^\top \mathbf {y}\le 1$ gives rise to a cp factorization (8);
3. else $\pi _0 >1$ (this includes infeasibility of (16) by putting $\pi _0 = + \infty$);

      then (11) has no solution.

(b1) Consider the convex quadratically constrained QP

17 $$\begin{aligned} \pi _1 = \min \left\{ \mathbf {y}^\top \mathbf {y}: \mathbf {y}\in {\mathbb {R}}^r \, , \, F^\top \mathbf {y}=\mathbf {x}\, ,\, \mathbf {y}\mathbf {x}^\top + (\mathbf {y}^\top \mathbf {y})F \le 2 F \right\} \, . \end{aligned}$$

Then

1. any feasible $\mathbf {y}$ with $\mathbf {y}\ge \mathbf {o}$ satisfies $\mathbf {y}^\top \mathbf {y}\le 1$;
2. any feasible $\mathbf {y}$ with $\mathbf {y}^\top \mathbf {y}\le 1$ gives rise to a cp factorization (8);
3. else $\pi _1 >1$ (this includes infeasibility of (17) by putting $\pi _1 = + \infty$);

      then (12) has no solution.

(c1) Consider the convex nonsmoothly constrained QP

18 $$\begin{aligned} \pi _e = \min \left\{ \mathbf {y}^\top \mathbf {y}: \mathbf {y}\in {\mathbb {R}}^r \, , \, F^\top \mathbf {y}=\mathbf {x}\, ,\, \left( [\mathbf {y}\mathbf {x}^\top -F]_+ \right) ^{\bullet 2} +(\mathbf {y}^\top \mathbf {y}) F^{\bullet 2} \le F^{\bullet 2} \right\} .\nonumber \\ \end{aligned}$$

Then

1. any feasible $\mathbf {y}$ gives rise to a cp factorization (8);
2. else (18) is infeasible, and (13) has no solution.

### Proof

Statements (a1) and (b1) follow by multiplying the inequalities in (16) and (17), respectively, by $\mathbf {y}^\top \ge \mathbf {o}$ from the left, observing $\mathbf {y}^\top F= \mathbf {x}^\top$. So only (c2) remains to be shown. Since $\mathbf {x}\ne \mathbf {o}$ by assumption, also $F^\top \ne O$. If $F_{ij}>0$, then (18)-feasibility of $\mathbf {y}$ implies $(\mathbf {y}^\top \mathbf {y})F_{ij}^2 \le F_{ij}^2$ and thus $\mathbf {y}^\top \mathbf {y}\le 1$. $\square$

Problem (18) can be rewritten as a convex quadratically constrained QP, introducing more variables and more (linear) constraints:

$$\begin{aligned} \pi _e =\quad \min \limits _{(\mathbf {y},Z)\in {\mathbb {R}}^r\times {\mathbb {R}}^{r\times n}_+}\quad \quad \left\{ \mathbf {y}^\top \mathbf {y}: F^\top \mathbf {y}{=} \mathbf {x}, \mathbf {y}\mathbf {x}^\top {-}F\le Z, Z^{\bullet 2} {+}(\mathbf {y}^\top \mathbf {y}) F^{\bullet 2} \le F^{\bullet 2} \right\} . \end{aligned}$$

## Some empirical evidence

If *F* is square (implying $\text{ cp-rank }(X)\le n$) and nonsingular, $\mathbf {y}=F^{-\top }\mathbf {x}$ is the unique solution to $F^\top \mathbf {y}=\mathbf {x}$ (here $F^{-\top }=(F^\top )^{-1}$). Then the problems (16),  (17) and (18) are of no use, and one can check directly whether or not $\mathbf {y}= \mathbf {p}$ satisfies (11), (12), or (13). In a small simulation study, 1600 such square *F* matrices with positive entries were generated randomly and $X=F^\top F$. For a parameter $\sigma \in \left\{ 1, 1.1, 2, 100 \right\}$, a vector $\mathbf {x}\in {\mathbb {R}}^n_+$ was drawn at random and rescaled such that $\mathbf {x}^\top X^{-1}\mathbf {x}= \frac{1}{\sigma }$. Obviously $\lambda _\mathrm{min} (X-\mathbf {x}\mathbf {x}^\top )$ increases with $\sigma$ while $\sigma =1$ means that the generated matrix $Y\in {\mathcal {P}}\cap {\mathcal {N}}$ is singular. It turns out that large values of $\sigma$ favour the proposed approach even for relatively large instances, but even in singular cases a success is often encountered, particularly for moderate dimensions ($n+1\in \left\{ 5,20,100 \right\}$ was chosen). Overall we observe successes in more than 63% of the cases, which increases to 77% if $\sigma \ge 2$. The details are given in Table 1. The four numbers in every cell count how often the conditions $\mathbf {y}\ge \mathbf {o}$, (11), (12), and (13) are met for $\mathbf {y}=\mathbf {p}$. The last column gives the success rate across all *n*, cumulated over all cases with $\mathbf {x}^\top X^{-1}\mathbf {x}\ge \frac{1}{\sigma }$.

**Table 1:** Success percentages for conditions $[\mathbf {y}\ge \mathbf {o}]|$(11)|(12)|(13) with $\mathbf {y}^\top \mathbf {y}= \frac{1}{\sigma }$. For every cell 100 random $(n+1)\times (n+1)$-instances were generated

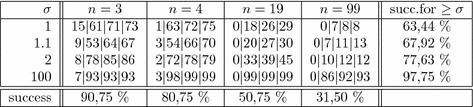	

The figures reported in Table 1 are quite encouraging. Remember that we generated matrices $Y\in {\mathcal {P}}\cap {\mathcal {N}}$ which have a completely positive block *X* with cp-rank not exceeding *n*, but we are not sure that *Y* is completely positive. So the decrease of success rates with increasing dimension have to be discounted by the probability that matrices *Y* of this kind are completely positive with cp-rank not exceeding $n+1$.

In the generation process, we did not use the construction as in Salce and Zanardo (1993) with nonnegative $s\times n$ matrix $F_0$ and $\mathbf {y}_0\in {\mathbb {R}}^s_+$ with $\mathbf {y}_0^\top \mathbf {y}_0 =1$, generating $X=F_0^\top F_0$ and $x= F_0^\top \mathbf {y}_0$ to ensure complete positivity of *Y*, for the following reason: then we either would have a trivial success for the simulation (if we choose *F* such that $F^\top ({\mathbb {R}}^r ) = F_0^\top ({\mathbb {R}}^s)$ like $F = F_0$ or $F^\top = [F_0^\top \, ,\,O]$ or $F^\top = [\frac{1}{\sqrt{2}}F_0^\top \, ,\,\frac{1}{\sqrt{2}}F_0^\top ]$), or else we would have to pick an essentially different *F* such that $F^\top F=X$, which is not obvious at all.

Now we turn to cp-ranks possibly exceeding dimension. Here, we must use the problem (6) and one of the problems (16),  (17), or (18). For simplicity, and because the differences in Table 1 were not that pronounced with the different approaches, we chose the convex, linearly constrained quadratic problem (16). The MatLab solvers linprog and quadprog were used. We basically follow the same experimental scheme, but for numerical reasons we restrict attention to the non-singular cases $\sigma \ge 1.1$. For dimension $n+1=5$, we allow for $m+1={n+1\atopwithdelims ()2}=10$ even larger than the maximal cp-rank, for dimension $n+1=20$ we use $(m+1)= (n+1)^2/4=100$ suggested by Drew et al. (1994), and for dimension $n+1=100$ we simply double $m+1=2(n+1)$. For smaller *n*, we also observed that increasing *m* in the range between 2*n* and $n^2/2$ increases success probability, so that the generated instances indeed are a priori not too easy for the proposed method. Nevertheless, the previously observed patterns persist and possibly get even more pronounced. Details can be found in Table 2. Finally it is worth mentioning that Kaykobad’s sufficient condition (Kaykobad 1987) of diagonal dominance was almost never met in both experiments; this diagonal dominance criterion follows from the results in Salce and Zanardo (1993), in a straightforward way, but appears to be too stringent a sufficient condition on average even for small dimensions. A similar statement holds for the PLSS property discussed shortly before Sect. 3.1.

**Table 2:** Success percentages for the problems (6)|(16) with $\mathbf {x}^\top X^+ \mathbf {x}= \frac{1}{\sigma }$. For every cell 100 random $(n+1)\times (n+1)$-instances were generated

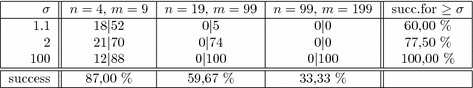	

The case of $n=4$ is particularly interesting, as we have ${\mathcal {C}}= {\mathcal {P}}\cap {\mathcal {N}}$ in dimension four, but strict inclusion in dimension 5. The papers (Berman and Xu 2004; Burer et al. 2009; Dong and Anstreicher 2010; Loewy and Tam 2003) discuss the $5\times 5$ case.

For illustration, we now specify five instances generated by above construction with $\sigma =2$. Recall in these cases always necessarily $\mathbf {y}=\mathbf {p}$, as *F* is square nonsingular.

$$\begin{aligned}{}[F_1|\mathbf {x}_1|\mathbf {p}_1]=\left[ \begin{array}{llll|l|l} 0.0245 &{}\quad 0.4916 &{}\quad 0.2664 &{}\quad 0.1435 &{}\quad 0.0334 &{}\quad 0.4044 \\ 0.0860 &{}\quad 0.9231 &{}\quad 0.8934 &{}\quad 0.6927 &{}\quad 0.0396 &{}\quad -\,0.3237 \\ 0.4181 &{}\quad 0.1568 &{}\quad 0.8756 &{}\quad 0.4079 &{}\quad 0.0598 &{}\quad -\,0.1253 \\ 0.2231 &{}\quad 0.3427 &{}\quad 0.7553 &{}\quad 0.5943 &{}\quad 0.0589 &{}\quad 0.4648 \\ \end{array}\right] \end{aligned}$$

satisfy (11) and therefore also (12) and (13), while

$$\begin{aligned}{}[F_2|\mathbf {x}_2|\mathbf {p}_2]=\left[ \begin{array}{llll|l|l} 0.6368 &{}\quad 0.8460 &{}\quad 0.8173 &{}\quad 0.3300 &{}\quad 0.6742 &{}\quad 0.5800\\ 0.7691 &{}\quad 0.1724 &{}\quad 0.2346 &{}\quad 0.2055 &{}\quad 0.6050 &{}\quad 0.3694\\ 0.0540 &{}\quad 0.0370 &{}\quad 0.5874 &{}\quad 0.3645 &{}\quad 0.7360 &{}\quad 0.0535\\ 0.1148 &{}\quad 0.3126 &{}\quad 0.9242 &{}\quad 0.9757 &{}\quad 0.4387 &{}\quad 0.1556\\ \end{array}\right] \end{aligned}$$

violate (11), but satisfy (12) and therefore (13). On the other hand,

$$\begin{aligned}{}[F_3|\mathbf {x}_3|\mathbf {p}_3]=\left[ \begin{array}{llll|l|l} 0.0743 &{}\quad 0.7709 &{}\quad 0.5029 &{}\quad 0.1131 &{}\quad 0.0536 &{}\quad 0.6108\\ 0.1932 &{}\quad 0.3139 &{}\quad 0.9477 &{}\quad 0.8121 &{}\quad 0.3349 &{}\quad 0.1541\\ 0.3796 &{}\quad 0.6382 &{}\quad 0.8280 &{}\quad 0.9083 &{}\quad 0.3169 &{}\quad 0.1500\\ 0.2764 &{}\quad 0.9866 &{}\quad 0.9176 &{}\quad 0.1564 &{}\quad 0.2861 &{}\quad -\,0.2840\\ \end{array}\right] \end{aligned}$$

violate (12) and therefore (11), but satisfy (13). Finally

$$\begin{aligned}{}[F_4|\mathbf {x}_4|\mathbf {p}_4]=\left[ \begin{array}{lll|l|l} 0.4154 &{}\quad 0.0150 &{}\quad 0.9901 &{}\quad 0.4921 &{}\quad 0.6135\\ 0.3050 &{}\quad 0.7680 &{}\quad 0.7889 &{}\quad 0.2113 &{}\quad -\,0.1430\\ 0.8744 &{}\quad 0.9708 &{}\quad 0.4387 &{}\quad 0.6355 &{}\quad 0.3213\\ \end{array}\right] \end{aligned}$$

violate (13) and therefore our methods presented up to now do not apply to $$Y_4 = \left[ \begin{array}{ll} 1 &{}\mathbf {x}_4^\top \\ \mathbf {x}_4 &{} F_4^\top F_4\\ \end{array}\right] $$.Since $Y_4\in {\mathcal {P}}\cap {\mathcal {N}}$ is a $4\times 4$-matrix, it must be completely positive, and in Sect. 7 we will factorize it by a refinement of these methods. The last example

$$\begin{aligned}{}[F_5| \mathbf {x}_5| \mathbf {p}_5]=\left[ \begin{array}{lll|l|l} 0.5369 &{}\quad 0.4175 &{}\quad 0.7538 &{}\quad 0.3648 &{}\quad 0.3416 \\ 0.0665 &{}\quad 0.2923 &{}\quad 0.0968 &{}\quad 0.3870 &{}\quad 0.5449 \\ 0.4939 &{}\quad 0.2897 &{}\quad 0.0769 &{}\quad 0.3329 &{}\quad 0.2938 \\ \end{array}\right] \end{aligned}$$

illustrates that the condition $\mathbf {p}\ge \mathbf {o}$ from Salce and Zanardo (1993) can be satisfied even if our condition (13) is violated, so that it really pays to combine all methods (although in higher dimensions, importance of the lower-triangular factorization decreases).

## Explicit factorization of low order matrices

We start by discussing the (doubly nonnegative) $2\times 2$ case. If a diagonal entry is zero, there is only one positive factorization (of rank zero or one). Else, we may and do rescale as before so that that $$X= \left[ \begin{array}{ll} 1 &{}\quad a \\ a &{}\quad 1\\ \end{array} \right] \in {\mathcal {P}}\cap {\mathcal {N}}$$. We may assume $0\le a <1$, because otherwise ($a =1$ and) $X= \mathbf {e}\mathbf {e}^\top$ with $\mathbf {e}^\top = [1,1]$. In this case, there is a factorization

19 $$\begin{aligned} X= F^\top F\qquad \text{ with } \qquad F= \left[ \begin{array}{ll} 1 &{}\quad a \\ 0 &{}\quad \sqrt{1-a^2}\\ \end{array}\right] \, . \end{aligned}$$

Now let us proceed to the three-dimensional case. We basically show that either of the factorizations (8) or (5) apply with the same *F*. So we again consider

20 $$\begin{aligned} Y =\left[ \begin{array}{ll} 1 &{}\quad \mathbf {x}^\top \\ \mathbf {x}&{}\quad X\\ \end{array}\right] \in {\mathcal {P}}\cap {\mathcal {N}}\, , \end{aligned}$$

which is equivalent to stipulate $X\in {\mathcal {P}}\cap {\mathcal {N}}$; $\mathbf {x}\in {\mathbb {R}}^2_+$; and $X-\mathbf {x}\mathbf {x}^\top \in {\mathcal {P}}$. Again we may and do assume $\text{ diag } X = \mathbf {e}=[1,1]^\top$, and $X=F^\top F$ with *F* as in (19). If *X* is singular, then $X=\mathbf {e}\mathbf {e}^\top$; the condition $\mathbf {e}\mathbf {e}^\top - \mathbf {x}\mathbf {x}^\top \in {\mathcal {P}}$ implies $\mathbf {e}=\alpha \mathbf {x}$ for some $\alpha \ge 1$ and

$$\begin{aligned} Y= \left[ \begin{array}{ll} 1 &{}\quad 0 \\ \mathbf {x}&{}\quad \sqrt{\alpha ^2-1}\,\mathbf {x}\\ \end{array}\right] \left[ \begin{array}{ll} 1 &{}\quad \mathbf {x}^\top \\ 0 &{}\quad \sqrt{\alpha ^2-1}\,\mathbf {x}^\top \\ \end{array}\right] \end{aligned}$$

is the cp factorization of the form (8). However, if *X* is nonsingular, so is *F* and $$F^{-\top }= \frac{1}{\sqrt{1-a^2}}\, \left[ \begin{array}{ll} \sqrt{1-a^2} &{}\quad 0 \\ -a &{}\quad 1\\ \end{array}\right] $$. So we get $y_1 = p_1= x_1 \ge 0$ whereas $y_2 = p_2 = \frac{x_2-ax_1}{\sqrt{1-a^2}}$ can have either sign.

Next we distinguish cases according to the sign of $y_2$. If $y_2\ge 0$, then $\mathbf {y}\in {\mathbb {R}}^2_+$ and we arrive at the factorization of the form (5):

21 $$\begin{aligned} Y = \left[ \begin{array}{ll} \mathbf {y}^\top &{}\quad \sqrt{1-\mathbf {y}^\top \mathbf {y}} \\ F^\top &{}\quad \mathbf {o}\\ \end{array}\right] \, \left[ \begin{array}{ll} \mathbf {y}&{}\quad F \\ \sqrt{1-\mathbf {y}^\top \mathbf {y}} &{}\quad \mathbf {o}^\top \\ \end{array}\right] \end{aligned}$$

If, however, $y_2 <0$, then the matrix $I_2 - \mathbf {y}\mathbf {y}^\top \in {\mathcal {P}}\cap {\mathcal {N}}$ (and this is true only if $\mathbf {y}$ has at most two nonzero entries of opposite sign, which is guaranteed only for $n=2$!), so we may factorize by taking square roots. Indeed, from (9) we have

$$\begin{aligned} Q = {[I_2 - \mathbf {y}\mathbf {y}^\top ]}^{1/2} = I_n - \beta \mathbf {y}\mathbf {y}^\top \qquad \text{ with }\qquad \beta = \frac{1}{\varphi (\mathbf {y}^\top \mathbf {y})} \le 1 \end{aligned}$$

which again has no negative entry in this particular case of order two when $y_1 y_2\le 0$. By consequence, and this time straighforwardly as both factors of *G* are nonnegative,

22 $$\begin{aligned} X-\mathbf {x}\mathbf {x}^\top = F^\top F - F^\top \mathbf {y}\mathbf {y}^\top F = F^\top \bar{X} F = G^\top G\,\text{ with }\,G= QF \; \text{ nonnegative. } \end{aligned}$$

So we obtain the desired factorization of the form (8). This establishes also the well-known fact that the cp-rank of any completely positive $3\times 3$ matrix is at most three. The elementary argument here differs from the more involved one in Berman and Shaked-Monderer (2003, Cor. 2.13, p. 126) which however establishes factorizations of triangular type whereas the above *F* is usually more dense, e.g. for the data of Berman and Shaked-Monderer (2003, Ex.2.19,p.126). For alternative, still different factorization results for all matrices of *rank* 3 (which can result in $r\ge 4$ if $n\ge 4$, cf. Berman and Shaked-Monderer (2003, Ex. 3.1, p. 140)) see Barioli and Berman (2003), Brandts and Křížek (2016).

## Towards larger cp-rank increments

Now, if we start with a cp-rank not exceeding *n*, as for $n=2$ or $n=3$, and apply the construction of Theorem 3.1 in a recursive way to building completely positive factorization, we can reach only matrices with the same property (cp-rank less or equal order). Indeed, *H* has $r+1$ rows if *G* has *r*, like *F*. The same is intrinsically true if we aim for the factorization (5).

Nevertheless, this still has some justification as the solutions of copositive optimization problems arising from most applications are expected to have a low cp-rank. An admittedly heuristic argument goes as follows: imitating the proof of Shaked-Monderer et al. (2015, Thm.3.4), one can show that for every matrix $Y\in {\mathcal {C}}$ one can construct a matrix $\widehat{Y}\in \partial {\mathcal {C}}$ with the same or larger cp-rank. But for a boundary point $\widehat{Y} = \widehat{H}^\top \widehat{H}$ we always can find a copositive matrix $S\in {\mathcal {C}}^*{\setminus } \left\{ O \right\}$ which is orthogonal to it, i.e., $trace(\widehat{Y} S)=0$. It follows that all columns of $\widehat{H}^\top$ must be global minimizers (with optimal value zero) of the quadratic form $\mathbf {x}^\top S \mathbf {x}$ over the standard simplex $\left\{ \mathbf {x}\in {\mathbb {R}}^n_+ : \sum _i x_i = 1 \right\}$. A recent study on this problem (Bomze 2017), corroborated by asymptotic probabilistic results (Chen and Peng 2015; Chen et al. 2013; Kontogiannis and Spirakis 2006, 2009, 2010) shows that very few *S* have many global minimizers (although in the worst case there can even coexist an exponential number of them).

Anyhow, we can extend the strategy of (22) towards larger cp-rank increments as follows if $X-\mathbf {x}\mathbf {x}^\top$ is completely positive. Note that below arguments do not rely on knowledge of the cp-rank. Indeed, instead of a minimal cp factorization of *X* we can start with any one. We will aim at enlarging the number of rows of *G* which will play the same role in *H* as in (8). As an aside, we note that by this construction, we get two alternative factorizations for *X*: the starting one, $X=F^\top F$ and the resulting one, $X=\mathbf {x}\mathbf {x}^\top + G^\top G = {\tilde{F}}^\top \tilde{F}$ where ${\tilde{F}}^\top = [\mathbf {x}\, |\, G^\top ]$ has one more column than $G^\top$, so the latter won’t be the minimal one if we succeed with our strategy. The same was already true for Theorem 3.1 where *H* had one more row than *F*.

First we explain why we can assume without loss of generality that for a solution $\mathbf {y}\in {\mathbb {R}}^n$ (with some negative entries) to $F^\top \mathbf {y}=\mathbf {x}$, we have equality $\Vert \mathbf {y} \Vert =1$:

### Proposition 7.1

Consider a solution $\mathbf {y}$ to $F^\top \mathbf {y}=\mathbf {x}$ with $\nu := \Vert \mathbf {y} \Vert < 1$ and define $\bar{\mathbf {y}}:=\frac{1}{\nu }\, \mathbf {y}$ as well as $\bar{\mathbf {x}} := \frac{1}{\sqrt{\nu }} \,\mathbf {x}$, $\bar{X} := \nu X$. Then

- if $X= F^\top F$, then $\bar{X} = \bar{F} ^\top \bar{F}$ where $\bar{F} := \sqrt{\nu }F\ge O$ satisfies $\bar{F}^\top \bar{\mathbf {y}} = \bar{\mathbf {x}}$ with $\Vert \bar{\mathbf {y}} \Vert =1$;
- from any cp factorization of $$\bar{Y} := \left[ \begin{array}{ll} 1 &{} \bar{\mathbf {x}}^\top \\ \bar{\mathbf {x}} &{} \bar{X}\\ \end{array}\right] = \bar{H}^\top \bar{H}$$ we get a cp factorization of *Y* as follows:
  $$\begin{aligned} Y = H^\top H \qquad \text{ with } \qquad H = \left[ \begin{array}{c} \sqrt{1-\sqrt{\nu }}\, \mathbf {e}_1^\top \\ \sqrt{\frac{1}{\nu }-\sqrt{\nu }}\,[\mathbf {o}\;\; \bar{F} ]\\ \nu ^{1/4} \bar{H} \\ \end{array}\right] \, . \end{aligned}$$

### Proof

(a) is straightforward. Likewise, to establish (b), we calculate

$$\begin{aligned} H^\top H = (1-\sqrt{\nu })\mathbf {e}_1\mathbf {e}_1^\top + \left( \frac{1}{\nu }- \sqrt{\nu }\right) \left[ \begin{array}{ll} 0 &{}\quad \mathbf {o}^\top \\ \mathbf {o}&{}\quad \bar{X}\\ \end{array}\right] + \sqrt{\nu } \,\bar{Y} =\left[ \begin{array}{ll} 1 &{}\quad \sqrt{\nu }\bar{\mathbf {x}}^\top \\ \sqrt{\nu }\bar{\mathbf {x}} &{}\quad \frac{1}{\nu } \bar{X}\\ \end{array}\right] = Y\,, \end{aligned}$$

observing in addition the alternative representation

$$\begin{aligned} \sqrt{\frac{1}{\nu }-\sqrt{\nu }}\,\bar{F} =\sqrt{1 - \nu ^{3/2}}F \end{aligned}$$

which can be used to build *H* when starting from the original factorization $X=F^\top F$, but $\bar{F}$ seems more natural as it occurs anyhow when building $\bar{H}$. $\square$

So let us assume in the sequel that $\Vert \mathbf {y} \Vert = 1$. The next step will imitate the case $y_1y_2<0$ in the previous section: decompose $\mathbf {y}^\top = [\mathbf {u}^\top \, |\,-\mathbf {v}^\top ]$ such that $\mathbf {u}\in {\mathbb {R}}^k_+{\setminus } \left\{ \mathbf {o} \right\}$ and $\mathbf {v}\in {\mathbb {R}}^m_+{\setminus } \left\{ \mathbf {o} \right\}$ with $k+m=r$ if $\mathbf {y}\in {\mathbb {R}}^r$, i.e., if *F* is an $r\times n$ matrix. As $\mathbf {u}^\top \mathbf {u}+ \mathbf {v}^\top \mathbf {v}= \mathbf {y}^\top \mathbf {y}= 1$ we thus have $0< \Vert \mathbf {u} \Vert <1$ and $0< \Vert \mathbf {v} \Vert < 1$, hence both $I_k - \mathbf {u}\mathbf {u}^\top$ and $I_m-\mathbf {v}\mathbf {v}^\top$ are positive-definite (but their square roots will have negative entries unless $k=1$ or $m=1$). We rescale $\mathbf {u}$ and $\mathbf {v}$ and imitate the high-cp-rank construction by Shaked-Monderer et al. (2013, Prop. 2.1): consider

23 $$\begin{aligned} Q:= \left[ \frac{\mathbf {v}}{ \Vert \mathbf {u} \Vert } \otimes {[I_k-\mathbf {u}\mathbf {u}^\top ]}^{1/2} \, | \, {[I_m-\mathbf {v}\mathbf {v}^\top ]}^{1/2} \otimes \frac{\mathbf {u}}{ \Vert \mathbf {v} \Vert } \right] \, , \end{aligned}$$

a matrix with $km\le r^2/4$ rows and $k+m=r$ columns. So, if the signs of entries of $\mathbf {y}$ are well balanced, this leaves a chance for a larger increment in cp-rank.

### Theorem 7.2

With above assumptions and notations, suppose that $F^\top F = X$ and $\mathbf {y}\in {\mathbb {R}}^r$ solves $F^\top \mathbf {y}= \mathbf {x}$ with $\Vert \mathbf {y} \Vert =1$. To obtain *Q* as in (23), decompose

24 $$\begin{aligned} \mathbf {y}= \left[ \begin{array}{c} \mathbf {u}\\ -\mathbf {v}\\ \end{array}\right] \quad \text{ with }\quad (\mathbf {u},\mathbf {v})\in {\mathbb {R}}^k_+\times {\mathbb {R}}^m_+\quad \text{ and }\quad F= \left[ \begin{array}{c} S \\ T\\ \end{array}\right] \end{aligned}$$

where *S* is a $k\times n$ matrix and *T* is an $m\times n$ matrix. Denote by $C:= I_k - \frac{\mathbf {u}\mathbf {u}^\top }{1+ \Vert \mathbf {v} \Vert }$ and $D:= I_m - \frac{\mathbf {v}\mathbf {v}^\top }{1+ \Vert \mathbf {u} \Vert }$. Then $C={[I_k-\mathbf {u}\mathbf {u}^\top ]}^{1/2}$ and $D= {[I_m-\mathbf {v}\mathbf {v}^\top ]}^{1/2}$.

Furthermore, let $\gamma = \frac{ \Vert \mathbf {v} \Vert }{ \Vert \mathbf {u} \Vert }$ and $$\Delta = \left[ \begin{array}{ll} \gamma I_k &{} O^\top \\ O &{} \frac{1}{\gamma }I_m\\ \end{array}\right] $$ and define $G:= Q\Delta ^{-1}F$. Then

- the condition $G\ge O$ can be rephrased as
  25 $$\begin{aligned} \frac{\mathbf {v}}{ \Vert \mathbf {v} \Vert }\otimes (CS) + (DT)\otimes \frac{\mathbf {u}}{ \Vert \mathbf {u} \Vert } \ge O\, , \end{aligned}$$
  while the condition $F^\top \mathbf {y}= \mathbf {x}$ amounts to $S^\top \mathbf {u}- T^\top \mathbf {v}= \mathbf {x}$.
- if $G\ge O$, then for the Schur complement we have $X- \mathbf {x}\mathbf {x}^\top = G^\top G$, so it is completely positive, too;
- the matrix
  $$\begin{aligned} H:=\left[ \begin{array}{ll} 1 &{}\quad \mathbf {x}^\top \\ \mathbf {o}&{}\quad G\\ \end{array}\right] \end{aligned}$$
  provides a cp factorization, $H^\top H = Y$;

### Proof

First recall (9) and that $\varphi (\mathbf {u}^\top \mathbf {u}) = 1+ \Vert \mathbf {v} \Vert$ due to $\mathbf {u}^\top \mathbf {u}+ \mathbf {v}^\top \mathbf {v}= { \Vert \mathbf {y} \Vert }^2 = 1$, which yields $C^2=I_k-\mathbf {u}\mathbf {u}^\top$ and likewise $D^2=I_m- \mathbf {v}\mathbf {v}^\top$. Next abbreviate by ${\mathbf {f}}= \frac{\mathbf {v}}{ \Vert \mathbf {u} \Vert }$ and by $\mathbf {g}= \frac{\mathbf {u}}{ \Vert \mathbf {v} \Vert }$. Then $Q= \left[ {\mathbf {f}}\otimes C \, | D \otimes \mathbf {g}\right]$ as defined in (23), and

$$\begin{aligned} C\mathbf {u}= \left( 1- \frac{{ \Vert \mathbf {u} \Vert }^2}{1+ \Vert \mathbf {v} \Vert }\right) \,\mathbf {u}= \frac{1+ \Vert \mathbf {v} \Vert - { \Vert \mathbf {u} \Vert }^2}{1+ \Vert \mathbf {v} \Vert } \, \mathbf {u}= \frac{(1+ \Vert \mathbf {v} \Vert ){ \Vert \mathbf {v} \Vert }}{1+ \Vert \mathbf {v} \Vert } \, \mathbf {u}= \Vert \mathbf {v} \Vert \, \mathbf {u}\, , \end{aligned}$$

so that we have $\mathbf {g}=C^{-1}\mathbf {u}$ and likewise ${\mathbf {f}}=D^{-1}\mathbf {v}$. To establish (a), tedious but straightforward calculations yield $Q\Delta ^{-1}F = \frac{1}{\gamma } {\mathbf {f}}\otimes (CS) + \gamma (DT)\otimes \mathbf {g}$, and therefore we arrive at (25). For (b), first note that by definition ${\mathbf {f}}^\top {\mathbf {f}}= \gamma ^2$ and $\mathbf {g}^\top \mathbf {g}= \frac{1}{\gamma ^2}$. Next recall that $C^\top \mathbf {g}= C\mathbf {g}= \mathbf {u}$ and $D^\top {\mathbf {f}}=D{\mathbf {f}}=\mathbf {v}$ by construction, so that we obtain

$$\begin{aligned} Q^\top Q= & {} \left[ \begin{array}{ll} ({\mathbf {f}}^\top \otimes C^\top )({\mathbf {f}}\otimes C) &{}\quad ({\mathbf {f}}^\top \otimes C^\top )(D\otimes \mathbf {g}) \\ (D^\top \otimes \mathbf {g}^\top )({\mathbf {f}}\otimes C) &{}\quad (D^\top \otimes \mathbf {g}^\top )(D\otimes \mathbf {g})\\ \end{array}\right] \\= & {} \left[ \begin{array}{ll} ({\mathbf {f}}^\top {\mathbf {f}})\otimes (C^\top C) &{}\quad ({\mathbf {f}}^\top D)\otimes (C^\top \mathbf {g}) \\ (D^\top {\mathbf {f}})\otimes (\mathbf {g}^\top C) &{}\quad (D^\top D)\otimes (\mathbf {g}^\top \mathbf {g})\\ \end{array}\right] \\= & {} \left[ \begin{array}{ll} \gamma ^2 C^2 &{}\quad \mathbf {u}\mathbf {v}^\top \\ \mathbf {v}\mathbf {u}^\top &{}\frac{1}{\gamma ^2} D^2\\ \end{array}\right] \\= & {} \left[ \begin{array}{ll} \gamma ^2(I_k - \mathbf {u}\mathbf {u}^\top ) &{}\quad \mathbf {u}\mathbf {v}^\top \\ \mathbf {v}\mathbf {u}^\top &{}\quad \frac{1}{\gamma ^2}(I_m-\mathbf {v}\mathbf {v}^\top )\\ \end{array}\right] \\= & {} \Delta (I_r - \mathbf {y}\mathbf {y}^\top )\Delta \qquad \text{ since }\quad \mathbf {y}\mathbf {y}^\top =\left[ \begin{array}{ll} \mathbf {u}\mathbf {u}^\top &{}\quad -\mathbf {u}\mathbf {v}^\top \\ -\mathbf {v}\mathbf {u}^\top &{}\quad \mathbf {v}\mathbf {v}^\top \\ \end{array}\right] . \end{aligned}$$

Hence $G^\top G = F^\top (I_r - \mathbf {y}\mathbf {y}^\top ) F = X - \mathbf {x}\mathbf {x}^\top$. (c) follows as before. $\square$

Similar strategies as in Sect. 4 can be employed to satisfy (25) which can be rephrased (with $\bar{\mathbf {u}} = \frac{\mathbf {u}}{ \Vert \mathbf {u} \Vert }$, $\bar{\mathbf {v}}= \frac{\mathbf {v}}{ \Vert \mathbf {v} \Vert }$) as

$$\begin{aligned} \bar{\mathbf {v}}\otimes S + T\otimes \bar{\mathbf {u}} \ge (\bar{\mathbf {v}}\otimes \bar{\mathbf {u}} ) \left[ \frac{1+ \Vert \mathbf {u} \Vert + \Vert \mathbf {v} \Vert }{(1+ \Vert \mathbf {u} \Vert )(1+ \Vert \mathbf {v} \Vert )}\, (T^\top \mathbf {v}) + \frac{ \Vert \mathbf {u} \Vert }{1+ \Vert \mathbf {v} \Vert }\,\mathbf {x}\right] ^\top \, . \end{aligned}$$

In case of square nonsingular *F*, the only choice for $\mathbf {y}=\mathbf {p}$, so the decomposition (24) is predetermined and above sufficient condition can be checked easily. Take for example the data $[F_4,\mathbf {x}_4]$ for $Y_4$ at the end of Sect. 5. After rescaling by $\nu = \Vert \mathbf {y}_4 \Vert =0.7070$ to $[\bar{F}_4,\bar{\mathbf {x}}_4]$ as in Proposition 7.1, we have

$$\begin{aligned} \bar{F}_4= & {} \quad \left[ \begin{array}{lll} 0.3493 &{}\quad 0.0126 &{}\quad 0.8325\\ 0.2565 &{}\quad 0.6458 &{}\quad 0.6633\\ 0.7352 &{}\quad 0.8163 &{}\quad 0.3689\\ \end{array}\right] \quad , \quad \left[ \begin{array}{c} \mathbf {u}\\ \mathbf {v}\\ \end{array}\right] \quad =\quad \left[ \begin{array}{c} 0.8676\\ 0.4544\\ 0.2021\\ \end{array}\right] \quad ,\\ G^\top \quad= & {} \quad \left[ \begin{array}{ll} 0.1120 &{}\quad 0.6110\\ 0.2973 &{}\quad 0.9654 \\ 0.7658 &{}\quad 0.3339 \\ \end{array}\right] \quad , \end{aligned}$$

which gives a cp factorization $$\bar{H} = \left[ \begin{array}{ll} 1&{}\quad \bar{\mathbf {x}}_4^\top \\ \mathbf {o}&{}\quad G\\ \end{array}\right] $$ of $\bar{Y}_4$, and from Proposition 7.1 we get

$$\begin{aligned} H^\top = \left[ \begin{array}{lllllll} 0.3990 &{}\quad 0 &{}\quad 0 &{}\quad 0 &{}\quad 0.9170 &{}\quad 0 &{}\quad \quad 0 \\ 0 &{}\quad 0.2645 &{}\quad 0.1942 &{}\quad 0.5568 &{}\quad 0.5367 &{}\quad 0.1027 &{}\quad 0.5602 \\ 0 &{}\quad 0.0096 &{}\quad 0.4891 &{}\quad 0.6182 &{}\quad 0.2304 &{}\quad 0.2726 &{}\quad 0.8852 \\ 0 &{}\quad 0.6305 &{}\quad 0.5024 &{}\quad 0.2794 &{}\quad 0.6930 &{}\quad 0.7022 &{}\quad 0.3062\\ \end{array}\right] \end{aligned}$$

which yields indeed $H^\top H = Y_4$.

Finally we briefly sketch a possible extension if Schur complements are no longer completely positive. Then we have to abandon the upper-triangular strategy and use a general

$$\begin{aligned} H= \left[ \begin{array}{ll} \alpha &{}\quad \mathbf {p}^\top \\ \mathbf {q}&{}\quad R\\ \end{array}\right] \quad \text{ with }\quad 0\le \alpha < 1\, , \end{aligned}$$

where

$$\begin{aligned} X-\mathbf {x}\mathbf {x}^\top = R^\top (I_n-\mathbf {q}\mathbf {q}^\top )R + (1-\alpha ^2)\mathbf {p}\mathbf {p}^\top - \alpha \left[ \mathbf {p}(R^\top \mathbf {q})^\top + (R^\top \mathbf {q})\mathbf {p}^\top \right] \, , \end{aligned}$$

which not necessarily is completely positive. Observe that the choice $[\mathbf {p}| R^\top ]=F^\top$ would lead back to solving $F^\top \mathbf {y}= \mathbf {x}$ with $\mathbf {y}= [\alpha , \mathbf {q}^\top ]^\top \in {\mathbb {R}}^r_+$. The difference is that, on one hand now ${ \Vert \mathbf {y} \Vert }^2 = \alpha ^2 + \mathbf {q}^\top \mathbf {q}= 1$ automatically by construction (but the previous $\Vert \mathbf {y} \Vert \le 1$ posed no restriction as we have seen in Proposition 7.1). On the other hand, now the north-west corner of *H* can be less than one, which opens more possibilities to proceed similarly as above. Given above empirical evidence, this may be a promising avenue of future research.
